# Temporal Arteritis Presenting as an Isolated Bilateral Abducens Nerve Palsy: A Rare Case of a 65-year-old Male

**DOI:** 10.7759/cureus.2667

**Published:** 2018-05-22

**Authors:** Abhishek Lunagariya, Chintan Rupareliya, Pradeep C Bollu, Zabeen Mahuwala

**Affiliations:** 1 Department of Neurology, University of California San Diego; 2 Department of Neurology, University Of Kentucky College of Medicine; 3 Department of Neurology, University of Missouri, Columbia, USA

**Keywords:** giant cell arteritis, temporal arteritis, abducens nerve palsy, panarteritis, sudden blindness, cranial nerve palsy, bilateral abducens nerve palsy, polymyalgia rheumatica, necrotizing granulomatous disease

## Abstract

Giant cell arteritis (GCA) or temporal arteritis (TA) is a granulomatous inflammation of medium to large-sized arteries. It may have a diverse presentation. The most common presenting symptoms of GCA are fever, malaise, unilateral headache, jaw claudication, polymyalgia rheumatica (PMR) and ophthalmoplegia. Most severe sequelae of GCA could be blindness. We report a case of a 65-year-old Caucasian male who presented for the third time with recurrent episodes of diplopia. Neurologic exam showed bilateral cranial nerve (CN) VI palsy, slightly worse on the right than the left side. Other focal neurological deficits were absent. GCA was considered and biopsy of the temporal artery was performed which showed necrotizing pan-arteritis, consistent with GCA. The patient was empirically treated with intravenous (IV) methylprednisolone while awaiting the biopsy results which resulted in the resolution of the symptoms. As far as we know, this is the second case in the literature about the bilateral sixth CN involvement in the background of GCA.

## Introduction

Abducens nerve palsy results in lateral rectus paresis and presents as binocular horizontal diplopia. It has multiple etiologies including trauma, vasculopathic lesion, inflammation, tumor, demyelinating disease, and sub-arachnoid hemorrhage [1]. Ophthalmoplegic manifestations of the giant cell arteritis (GCA) or temporal arteritis (TA) are rare and they are all vascular in origin. GCA is a chronic, systemic, inflammatory vasculitis affecting medium and large-sized arteries. Ocular manifestations of GCA may range from ischemia of optic nerve, retinal infarction, paresis of cranial nerves (CNs) supplying to eye muscles (CN III, IV and/or VI), pupillary autonomic dysfunction, posterior chiasmal field defects and cortical blindness [2].

Prevalence of GCA is 15 to 30 per 100,000 patients with most affected patients being 50 years and older. Most cases are seen in Caucasians with north European descents or North Americans, however, GCA may also affect Asians and other races [3]. Even though it is rare, sixth nerve palsy can be a potential manifestation of GCA. Based on the literature review, CN mononeuritis, bilateral third nerve involvement, and multiple CN involvement have been reported [1-4]. We describe a case which presented with isolated bilateral abducens nerve palsy that was found to have GCA after extensive workup and showed improvement with intravenous (IV) glucocorticoids treatment.

## Case presentation

**Figure 1:**
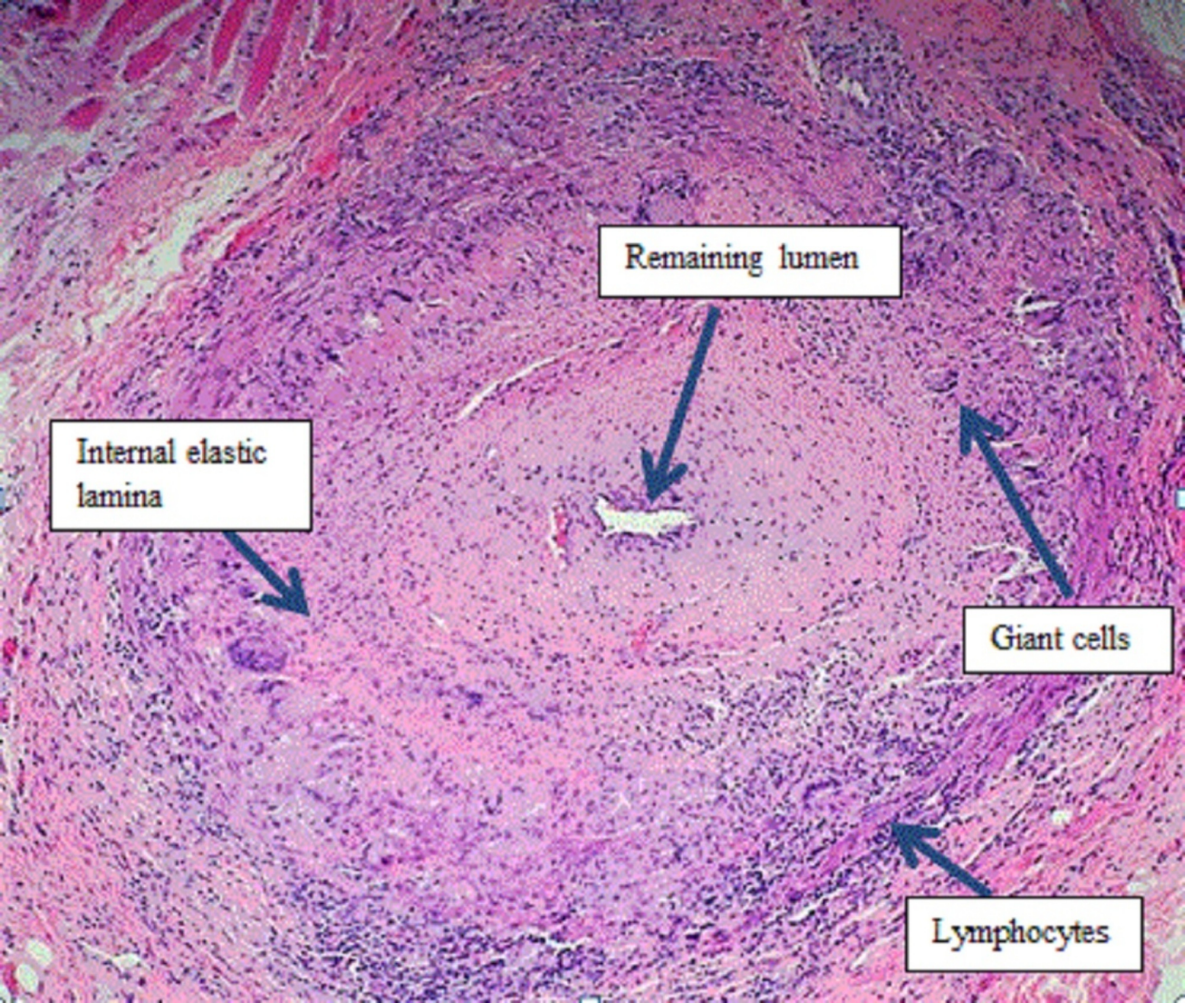
High-power hematoxylin & eosin stained section of the superficial temporal artery biopsy specimen revealing pan-arteritis with giant cells

A 65-year-old right-handed Caucasian male presented with recurrent episodes of diplopia. Prior two episodes were brief and self-resolving; however, the current episode started two months ago and progressively worsened. He started perceiving objects brighter in the right eye. He also reported malaise, shoulder pain bilaterally, and approximately 15 lbs weight loss in the last few months. Exam showed bilateral CN VI palsy (right more than left) without any other focal neurological finding. Myasthenia gravis was earlier suspected and was ruled out clinically by negative blood tests and electromyography. Endocrine workup including thyroid panel was unremarkable. Computerized tomography (CT) angiogram of the head and neck did not show any flow limiting vessel stenosis. Magnetic resonance imaging (MRI) of the brain did not show any diffusion restriction. Cerebrospinal fluid (CSF) analysis for infectious and inflammatory processes and serum autoimmune workup was negative. Occult malignancy was excluded by a whole body CT. Erythrocyte sedimentation rate (ESR) and c reactive protein (CRP) was remarkably elevated. Due to concerns of generalized malaise, weight loss, and elevated inflammatory markers in an elderly individual; TA was considered. A patient was empirically started on 1 g IV methylprednisolone (solumedrol) for three days followed by a gradual taper while waiting for the temporal artery biopsy results. Temporal artery biopsy showed necrotizing pan-arteritis consistent with GCA (Figure 1). The patient was discharged on 60 mg prednisolone. At one and two-month follow-up, the patient had improvement in diplopia and steroids were tapered off.

## Discussion

We report a case of histologically proven GCA that had an atypical presentation with bilateral CN VI paresis. Patients with GCA usually present with a unilateral headache, fever, musculoskeletal impairment owing to polymyalgia rheumatica (PMR) and visual symptoms [1, 3-9]. In our case, the patient had no headache or other focal neurological findings. His diplopia was episodic with the previous two episodes resolving on their own. In contrast to most GCA presentations which are ophthalmic emergencies, our case showed progressive worsening of diplopia during the third episode which proved to be CN VI paresis upon neurological examination.

Ocular CN involvement upon presentation, without the absence of other typical symptoms, suggesting GCA may lead to a complex process of differential diagnosis. Differential diagnosis in our case included myasthenia gravis, thyroid dysfunction, mass lesion inside the brain compressing the sixth CN, occult malignancy, and autoimmune inflammatory process. The American College of Rheumatology (ACR) had established the criteria in 1990 for the diagnosis of GCA which is shown in Table 1 [5]. A patient with vasculitis is said to have TA if at least three out of the five criteria are met. Additionally, one population-based study attempting to find out the relationship between GCA and PMR showed that the latter is present in around 40%-60% patients with GCA and about 16%-21% patients with PMR have GCA [6-8]. Another study revealed only 93.5% sensitivity and 91.2% specificity of ACR criteria for detecting GCA [9]. Our patient had two criteria meeting the guidelines which called for the temporal artery biopsy that confirmed the presence of GCA.

**Table 1:** Diagnostic criteria for GCA by the American College of Rheumatology (ACR) GCA: giant cell arteritis; ESR: elevated sedimentation rate; mm: millimeter.

Criteria	Details
Age > 50 years	Symptoms begin at the age of 50 years and/or older
Headache	New onset of localized pain in the head
Raised ESR	ESR > 50 mm/h by Westergren method
Temporal artery abnormality	Tenderness over the area of temporal artery on palpation and/or decreased pulsation
Temporal artery biopsy	Temporal artery biopsy showing vasculitis (granulomatous inflammation with multinucleated giant cells)

We did not wait for the biopsy results to begin the treatment as GCA is an ophthalmologic emergency with poor prognosis and irreversible visual loss being the most dreaded complication. One study that aimed at monitoring the visual symptoms improvement in GCA patients with corticosteroid therapy showed that 96% of GCA patients with visual loss did not improve even after corticosteroid therapy [10]. In our patient, the temporal artery biopsy results confirmed the diagnosis of GCA; the patient also remained in the constant remission after beginning the treatment.

GCA carries a broad range of presentation. Cases of GCA affecting different CNs have been reported before and include unilateral abducens nerve [11], bilateral abducens nerve [12], unilateral abducens with oculomotor nerve palsy [3], isolated eighth CN palsy [13-15], and eighth CN involvement with cough, conjunctivitis, and pulmonary nodules [16]. There were also cases of left recurrent laryngeal palsy with aortic arch aneurysm [17-18], hoarseness of voice [19-20] and simultaneous involvement of three CNs [4].

## Conclusions

GCA is a necrotizing and granulomatous inflammation of the medium and large size blood vessels. Untreated, it can result in permanent blindness. Though headache is a common association, it sometimes may be absent. Our case emphasizes the fact that GCA can present in an atypical way and that physicians should have a high level of suspicion as a prompt institution of treatment is necessary to prevent potential permanent blindness.
